# Validated RP‐HPLC‐UV Method for Simultaneous Quantification of Aminophenazone, Dipyrine, and Antihistamine in Pharmaceutical Formulations Considering Dipyrine Decomposition

**DOI:** 10.1155/ianc/7435313

**Published:** 2026-01-31

**Authors:** Md. Abid Hasan, Sajia Azmi, Tasnuma Tabassum, Ejaj Sumit, Abdul Gafur, Naima Helal

**Affiliations:** ^1^ Department of Pharmacy University of Asia Pacific Dhaka 1205 Bangladesh uap-bd.edu; ^2^ Department of Pharmacy University of Science and Technology Chittagong Chattogram 4202 Bangladesh ustc.ac.bd; ^3^ Department of Pharmacy University of Dhaka Dhaka Bangladesh du.ac.bd; ^4^ Department of Pharmacy East West University Dhaka Bangladesh ewubd.edu; ^5^ Department of Pharmacy Stamford University Bangladesh Dhaka Bangladesh stamforduniversity.edu.bd; ^6^ Department of Pharmacy World University of Bangladesh Dhaka 1230 Bangladesh wub.edu.bd

**Keywords:** aminophenazone, chlorpheniramine maleate, chromatography, dipyrine, validation

## Abstract

Aminophenazone, dipyrine, and chlorpheniramine maleate combined drug is used as antipyrine and analgesic. In this study, a rapid, robust, and straightforward high‐performance liquid chromatographic (HPLC) technique was developed, optimized, and validated for simultaneous analysis of aminophenazone, dipyrine, and chlorpheniramine maleate taking into account decomposition of dipyrine. On a C18 (4.6 mm × 15 cm; 5 μm) Shim‐pack column, aminophenazone, dipyrine, and chlorpheniramine maleate were successfully eluted by using mobile phase, comprising water: methanol: triethylamine: glacial acetic acid (70:28:1:1 v/v/v/v) at flow rate 1.0 mL/min and wavelength of 254 nm. About 5 mg/mL sodium sulfite in diluent and mobile phase was used to prevent the hydrolysis of dipyrine after several investigations. The validation parameters, including specificity, linearity, LOD/LOQ, precision, accuracy, robustness, and solution stability, were verified for performance of the method. In specificity study, there was not any interference with main peak. The constructed calibration curve was found to be linear in the concentration ranges of 0.1–1.0 (aminophenazone), 0.1–1.0 (dipyrine), 0.002–0.020 (chlorpheniramine maleate) mg/mL, respectively. The % recovery at different concentrations was within the (98.0–102.0) %. When the column temperature, the flow rate, wavelength, and mobile phase composition were changed, the absolute difference of the content at different modified conditions were within 2.0 (%RSD) of the original condition. The solution was stable upto 24 h in room temperature (benchtop), autosampler (15°C–25°C), and refrigerator (2–8 degree). This method was successfully employed for the analysis of aminopyrine, metamizole, and chlorpheniramine maleate in marketed products, and the results were satisfactory. The confirmed RP‐HPLC‐UV method may be a workable analytical approach on a routine analysis.

## 1. Introduction

Formulations with one or more active pharmaceutical ingredients (APIs) designed for usage in a fixed‐dosage form are known as medicinal combinations. Most multicomponent dosage forms have two or more active ingredients, each of which enhances the drug’s total efficacy for therapy. This concept is useful when the chosen drugs have different ways of working that result in additive or synergistic efficacy [1].

Drug manufacturers are looking for combination dose forms that can address several symptoms at once. Combining aminophenazone, dipyrine, and chlorpheniramine maleate is a common combination dosage used to treat viral disorders that cause fever or pain because of its analgesic, antipyretic, and antihistamine properties. This combination is used for analgesic and antipyretic effects on animals (cat, dog, goat, horse, and cattle) in many developing countries [2–4]. The Directorate General of Drug Administration (DGDA) [5] and Ministry of Food and Drug Safety [6] approved this combination drug for animal use only.

The over‐the‐counter medication aminophenazone (Figure 1(a)) (4‐dimethylamino‐2,3‐dimethyl‐1‐phenyl‐3‐ pyrazolin‐5‐one) has been widely used for decades since the 1990s due to its analgesic, antipyretic, and anti‐inflammatory actions [7]. It is still used in “herbal” medications to treat sick people in many countries, particularly China, even though it is no longer licensed for use in the majority of nations due to the danger of agranulocytosis [8].

FIGURE 1Chemical structure of (a) aminophenzone, (b) dipyrine, and (c) chlorpheniramine maleate.

**Figure:**
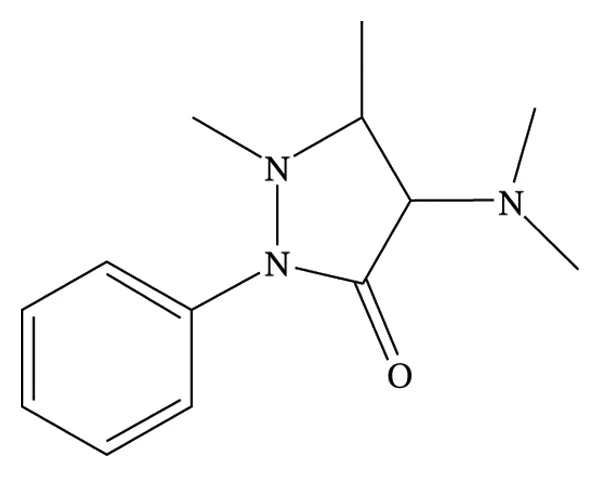
(a)

**Figure:**
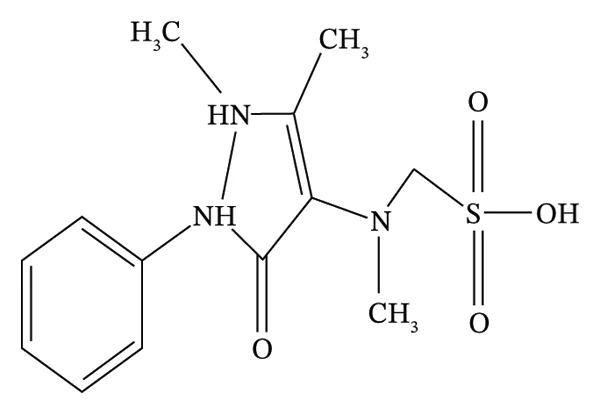
(b)

**Figure:**
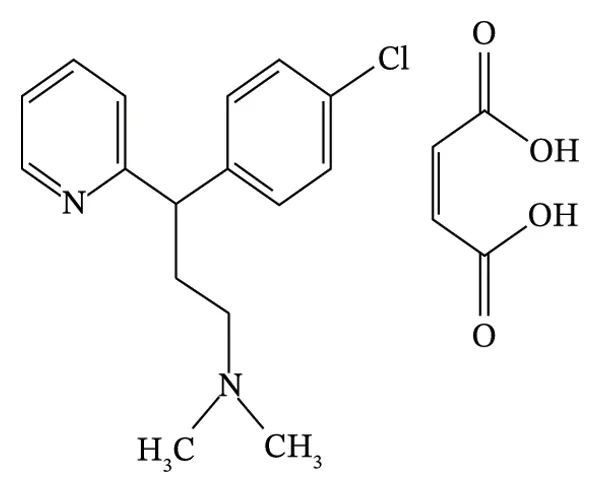
(c)

Dipyrine (Figure 1(b)) is a pyrazolone nonsteroidal anti‐inflammatory medication (NSAID) that is sodium [N‐(1,5‐dimethyl‐3‐oxo‐2‐phenylpyrazolin‐4‐yl)‐N‐methylamino]. Dipyrine exhibits strong antipyretic and analgesic effects. Its formulations can be used as an adjuvant therapy for a variety of inflammatory disorders affecting the musculoskeletal and locomotor systems in horses, cattle, and pigs [9–11]. Dipyrine hydrolyzes quickly and without the aid of enzymes to produce 4‐methylaminoantypirine (4‐MAA), its primary metabolite. The breakdown process is unimolecular [12–14]. The analgesic effect has been correlated to the concentration of 4‐MAA [15].

A first‐generation alkylamine sedating antihistamine that produces a modest level of drowsiness, chlorpheneramine maleate (Figure 1(c)) (CPM; 2‐[p‐chloro‐[2‐dimethylamino) ethyl] benzyl] pyridine maleate) is used to alleviate the symptoms of allergic diseases [16]. Both during absorption and its initial passage through the liver, it is significantly metabolized in the GI mucosa [17].

The goal of the research was to create a cost‐effective RP‐HPLC technique suitable for the routine simultaneous analysis of aminophenazone, dipyrine, and chlorpheniramine maleate present in pharmaceutical formulation that would be quick, inexpensive, and process efficient. Pharmaceutical items must undergo both qualitative and quantitative testing of their dosage forms to ensure quality. Quantitative testing needs to be done at every stage of the formulation product’s lifespan to guarantee stability and safety.

Various analytical methods (RP‐HPLC, LC‐MS‐MS, UV‐Spectroscopy, TLC, electrochemical, capillary electrophoresis, and spectrophotometric) are reported for the determination of aminophenazone [18–21], dipyrine [22–31], chlorpheniramine maleate [21, 32–45] either alone or combined with another drug.

According to a literature study, there are no analytical methods reported for simultaneous detection of aminophenazone, dipyrine, and chlorpheniramine maleate in pharmaceutical formulation in a single run. There is not a single accurate, reliable, and exact method for determining the level of aminophenazone, dipyrine, and chlorpheniramine maleate in parenteral dosage formulation in terms of both quality and quantity.

The goal of this work was to develop a single analytical technique that would enable the simultaneous measurement of aminophenazone, dipyrine, and chlorpheniramine maleate in injection formulation in a straightforward, repeatable, linear, stable, robust, and accurate manner. Based on a comprehensive review of the literature, the current approach is the first method ever developed for the quick and easy detection of aminophenazone, dipyrine, and chlorpheniramine maleate in injection formulations.

## 2. Materials and Methods

### 2.1. Instrument

Shimadzu HPLC system with SPD‐20A‐UV/Vis detector, and Waters e2695 (Waters Corporation, Milford, Ma, USA) HPLC system with 2489 UV/Vis detector, quaternary pump, vacuum degas, 100 μL loop, 120 vials were utilized for the project. Lab solution and Empower 3 were used for processing and evaluating the obtained data. Chromatographic column for separation was used Shim‐pack GIST, Japan. Ultrasonic Bath was Hwashin (model: powersonic 620), Korean brand. Analytical balance for weighing the chemicals and reagents was Mettler Toledo (model: MR304), Switzerland brand. pH meter was Mettler Toledo (model: S400), Switzerland brand.

### 2.2. Chemical and Reagent

Reagents were used from different reliable resources. Aminophenazone (Potency: 99.87%), dipyrine (Potency: 99.14%) API purchased from Zhejiang Haisen Pharmaceutical Company limited, China, and chlorpheniramine maleate (Potency: 99.56%) API was purchased from Supriya Life Science Pvt Limited, India. API used as working standard after standardization. The marketed products, (i) TD Pyrin Injection (each ml contains aminophenazone 50 mg, dipyrine 50 mg, and chlorpheniramine maleate 1 mg), manufacturer; Techno Drugs Limited and (ii) Samu Pyrin Injection (each ml contains 50 mg aminophenazone, 50 mg dipyrine, 1 mg chlorpheniramine maleate) manufacturer; Samu Median Co. Ltd, Korea, were purchased from a local pharmacy. Analytical grade reagent, methanol (Lot: I1267907654), was purchased from Merck, USA. Triethylamine (Batch: 23852906), sodium sulfite (Batch:20997502) from Scharlab and Glacial acetic acid (Lot: K55767963 555) from Merck, USA, were used throughout the study. Milli‐Q was used to obtain purified water.

### 2.3. Analytical Solution Preparation

#### 2.3.1. Mobile Phase

A mixture of water, methanol, triethylamine, and glacial acetic acid at a ratio of 70:28:1:1 v/v/v/v was prepared.

#### 2.3.2. Diluent

All solution preparation process uses the mobile phase as a diluent.

#### 2.3.3. Standard Solution

100 mg of aminophenazone, 100 mg of dipyrine, and 2 mg of chlorpheniramine maleate were transferred into a 50‐mL volumetric, respectively, to create stock solution. 5 mL of stock solution was transferred into 20 volumetric flasks to create a 0.5‐mg/mL aminophenazone, 0.5 mg/mL dipyrine, and 0.01 mg/mL chlorpheniramine maleate solution.

#### 2.3.4. Sample

About 2 mL of sample solution by weight (which is equivalent to 100 mg of aminophenazone, 100 mg of dipyrine, and 2 mg of chlorpheniramine maleate) was transferred to a 50‐mL volumetric flask. Add 30 mL of diluent and sonicate for about 5 min. Cool to room temperature. Make the volume up to the mark with diluent. Transfer 5 mL of the solution to a 20‐mL volumetric flask, volume with diluent. The concentration of API is the same as the standard solution.

### 2.4. Chromatographic Condition

The separation was done with C18; 4.6 mm × 15 cm; 5 μm, Shim‐pack column, and peak was detected at 254 nm wavelength. The injection volume was 10 μL, the temperature of the column oven was 30℃, and the total flow rate was 1.0 mL/min.

### 2.5. Analytical Method Validation

The approach was verified in compliance with ICH guidelines ICH‐Q2 (R1) [46] and United States of Pharmacopeia (USP) general chapter 1225 [47] with respect to system suitability, specificity, LOD/LOQ, linearity, precision, accuracy, robustness, and solution stability.

#### 2.5.1. System Suitability

The HPLC technology was assessed to confirm its reproducibility and compatibility for the planned analytical application. To evaluate this, the USP theoretical plate count, tailing factor, and relative standard deviation (RSD) from five injections of known concentration of standard solutions were looked at. The %RSD of aminophenazone, dipyrine, and chlorpheniramine maleate peak area must be within the 2.0, tailing factors not more than 2.0, and USP theoretical plate must be not less than 2000.

#### 2.5.2. Specificity

When the analyte peak can be clearly distinguished from other peaks, specificity has been verified. To check the interference of diluent with aminophenazone, dipyrine, and chlorpheniramine maleate, a specificity study was carried out. The separate 0.5 mg/mL aminophenazone, 0.5 mg/mL dipyrine, and 0.01 mg/mL chlorpheniramine maleate working standard and sample solution were prepared and injected individually. The chromatogram produced from the diluent should not have any interfering peaks at the retention time that corresponds to the peak for aminophenazone, dipyrine, and chlorpheniramine maleate. The retention time of peak due to aminophenazone, dipyrine, and chlorpheniramine maleate in sample solution shall be similar (±2%) to that of the standard.

#### 2.5.3. LOD/LOQ

The limit of detecting and the limit of quantifying have been calculated using the signal to noise (S/N) ratio technique. Aminophenazone, dipyrine, and chlorpheniramine maleate standard solution were prepared at different low‐level of concentration as much as possible and injected. The equation LOD = 3.3 *σ*/s and LOQ = 10 *σ*/s were used to determine the limits of detection and quantitation, respectively, where S is the calibration curve’s slope (as determined by the linearity curve) and σ is the response’s standard deviation.

After determination of LOD and LOQ concentration, LOD and LOQ solutions were prepared and injected. In the case of LOQ, S/N ratio of aminophenazone, dipyrine, and chlorpheniramine maleate peaks must be greater than or equal to 10. The peak at the LOD level should be detectable and S/N ratio must be more than 3.

#### 2.5.4. Linearity

Linearity of aminophenazone, dipyrine, and chlorpheniramine maleate was demonstrated by injecting standard solution of aminophenazone, dipyrine, and chlorpheniramine maleate in concentration from the LOQ concentration level to 200% of 100% concentration of the test sample (working concentration). The correlation coefficient (*r* ^2^), intercept (a), and slope (b) were determined by charting the peak area against the analyte concentration using a calibration curve. The r^2^ must not be less than 0.995. Besides, statistical parameters such as RSD of slope (Sb%), variables around the slope (Sb2) for different calibration data were performed [48–51]. To determine if the observed intercept (a) of the above regression lines was not different significantly from the null hypothesis, the paired *t*‐test must be used. The calculated t (a/(Sa)) values must not be more than the 95% criterion of t = 2.31 for five separate measurements. Consequently, the hypothesis that (a) has little significance was validated. High *F*‐value (low significance *F*) regression lines are superior to those with lower than *F*‐values. High values for both (*r*) and (*F*) values are indicated by good regression lines [49–51].

##### 2.5.4.1. Linearity Stock Solution Preparation

2.5 mg/mL of aminophenazone, 2.5 mg/mL of dipyrine, and 0.05 mg/mL of chlorpheniramine maleate stock solution were prepared in a 50‐mL volumetric flask. A series of concentration from stock linearity solution was prepared, as shown in (Table 1).

**TABLE 1:** Preparation of linearity solutions (20%–200%).

% Concentration	Volume taken from linearity stock solution (mL)	Total volume with diluent (mL)	Concentration (mg/mL)		
			Aminophenazone	Dipyrine	Chlorpheniramine maleate
20	2	50	0.1	0.1	0.002
50	2	20	0.25	0.25	0.005
80	4	25	0.4	0.4	0.008
100	4	20	0.5	0.5	0.01
120	6	25	0.6	0.6	0.012
200	4	10	1	1	0.02

#### 2.5.5. Precision Study

##### 2.5.5.1. Method Precision/Repeatability Study

Assess the repeatability by performing six replicate measurements (*n* = 6) of the assay of the sample as per test method at 100% of test concentration (0.5 mg, 0.5 mg, and 0.01 mg per mL) and figuring out the aminophenazone, dipyrine, and chlorpheniramine maleate area %RSD. The %RSD of six replicate areas of aminophenazone, dipyrine, and chlorpheniramine maleate must not be more than 2.0.

##### 2.5.5.2. Intermediate Precision Study

The intermediate precision was done by another analyst with different HPLC on a different day in another laboratory. Cumulative %RSD of each injection from 12 results of the sample (MP and IP) should be not more than 10.0%.

#### 2.5.6. Accuracy

The degree of agreement between the actual quantity and the test result indicates the method’s accuracy. To establish the accuracy, prepared spiked sample solution in triplicate by spiking aminophenazone, dipyrine, and chlorpheniramine maleate API and placebo at LOQ, 80%, 100%, and 120% of the working concentration (100%). The recovery (%) should be between 98.00% and 102.00%.

#### 2.5.7. Robustness

By evaluating minor intentional adjustments to the values in the technique development parameters, the stability of the optimized approach was examined. The standard solution and sample at test concentration were prepared and injected. Inject the prepared solution using different chromatographic conditions (a) by changing the column oven temperature by ±5℃ from the actual column oven temperature (30℃), (b) by changing the flow rate by ±0.2 mL/min from the actual flow rate (1.0 mL/min), and (c) mobile phase composition, organic composition ±5%.

The absolute difference of the content at different modified conditions was within 2.0 of the original condition.

#### 2.5.8. Solution Stability

To conduct solution stability, the standard and sample solutions were kept in room temperature (benchtop), autosampler (15℃ to 25℃), and refrigerator (2–8℃) for 24 h using both clear and amber vial. After 6, 12, and 24 h, all solutions were injected and compared with the initial results. The absolute difference between initial and different time interval for standard and sample solution should be within 2.0.

#### 2.5.9. Forced Degradation

Forced degradation research was carried out by subjecting the standard solution and drug product samples to a range of stress conditions, including acid/base hydrolysis, oxidation, photodegradation, and thermal stress. Table 2 shows the time and conditions. Periodically, stressed samples were examined; associated peaks were examined for separation factors, spectra purity, retention durations, and peak interference.

**TABLE 2:** Forced degradation conditions.

Stress type	Conditions	Time
Acid hydrolysis	1 N HCl; at 40°C	2 days
Base hydrolysis	0.02 N NaOH; at RT	2 h
Oxidation	0.2% H_2_O_2_ at 40°C; protected from light	7 days
Thermal	75°C	14 days
Photodegradation	UV light	3 days

#### 2.5.10. Statistical Analysis

Microsoft excel was used for statistical analysis and linearity study. All data are shown as mean ± standard deviation.

## 3. Results and Discussion

### 3.1. Challenge With Rapid Decomposition of Dipyrine

The main challenge was preventing the hydrolysis of dipyrine to 4‐MAA as the dipyrine undergoes rapid hydrolysis to the active moiety 4‐MAA.

Dipyrine acts as a pro‐drug which hydrolyzes rapidly to 4‐MAA [52–54]. This change happens in methanol as well as water, although it happens more quickly in water and at acidic pH levels [53]. 4‐MAA and dipyrine have similar pharmacologic actions. Since it is not fully digested by enzymes five minutes after oral intake, dipyrine works in the form of 4‐MAA [53]. In order to stop dipyrine from hydrolyzing, sodium sulfite (Na2SO3) had to be added to all dipyrine containing solutions as well as the mobile phase. This was necessary because tests revealed that dipyrine hydrolyzed to 4‐MAA even during the chromatographic process if sodium sulfite was not added [23, 31, 54]. We may compute the dipyrine and 4‐MAA peak while taking detector response variables into account because 4‐MAA exhibits the same pharmacologic effects as dipyrine [23, 31], but here we have tried to overcome the hydrolysis process.

Firstly, we have added sodium sulfite to the solution containing dipyrine without adding in the mobile phase, after the peaks of dipyrine at about 8 min, a peak attributed to 4‐MAA was added to the chromatogram (Figure 2(a)). So, sodium sulfite is required both in mobile phase and solution to prevent hydrolysis.

FIGURE 2Chromatogram of (a) standard solution with adding sodium sulfite in diluent but not in mobile phase; (b) standard solution with sodium sulfite in both diluent and mobile phase (concentration: 0.5 mg/mL); (c) standard solution with sodium sulfite in both diluent and mobile phase (concentration: 1 mg/mL); (d) standard solution with sodium sulfite both diluent and mobile phase (concentration: 2 mg/mL).

**Figure:**
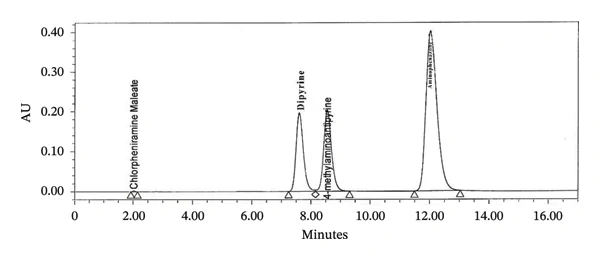
(a)

**Figure:**
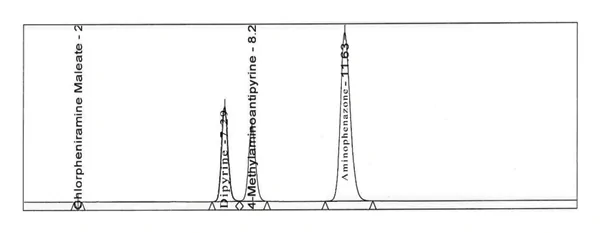
(b)

**Figure:**
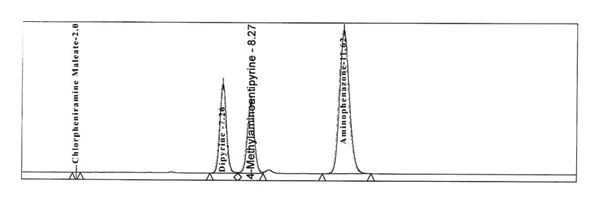
(c)

**Figure:**
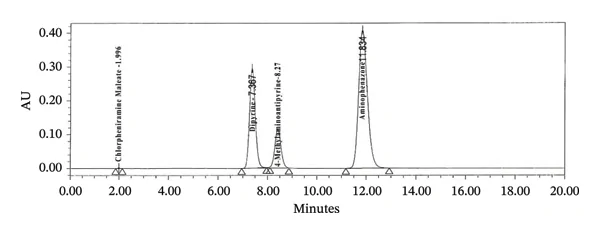
(d)

To determine the ideal concentration, the concentration of Na_2_SO_3_ in the mobile phase and solution was evaluated between 0.5 and 10 mg/mL (Figure 2(b), 2(c), and 2(d)). After several investigations, for the concentration use of dipyrine (0.5 mg/mL) in the solution, tests showed that 5 mg/mL Na_2_SO_3_ in mobile phase and solution were enough to prevent their hydrolysis. Thus, this concentration of Na_2_SO_3_ was utilized in the dipyrine and mobile phase solutions.

Concentrations higher than 5 mg/mL gave the same response chromatographic peak. Therefore, 5 mg/mL was selected as Na_2_SO_3_ appropriate dose to include in the mobile phase and solution for prevent dipyrine from hydrolysis.

### 3.2. Method Development and Optimization

In this study, the chromatographic method for simultaneous detection of aminophenazone, dipyrine, and chlorpheniramine maleate was carefully optimized. The purpose of the initial trials was to determine the optimum conditions including solvent, mobile phase composition, stationary phase, detection wavelength, and standard/sample concentration with preparation procedure, based on the physical and chemical properties of the aminophenazone, dipyrine, and chlorpheniramine maleate and the information gathered from the literature.

Several buffers in different proportions used as diluent and mobile phase were tested but the results were not satisfactory. After several attempts, the mobile phase, water, and methanol with triethylamine and glacial acetic acid produced an impressive peak and a steady baseline. Methanol was utilized as an organic solvent. In reverse phase HPLC, acetic acid and triethylamine, two conventional mobile phase additions, were used to provide acidic and alkaline conditions, respectively. Initially, the mobile phase was chosen as a 50:50 v/v mixture of water and methanol based on the physicochemical parameters of the analytes (aminophenazone and chlorpheniramine maleate are soluble in water, dipyrine soluble in methanol). The first attempt at simultaneously eluting the analytes under these isocratic chromatographic conditions has been successful; the column efficiency and aminophenazone, dipyrine, and chlorpheniramine maleate peak symmetry were both satisfactory, but the peak eluted too early. This required altering the composition of the mobile phase. Following investigation of water and methanol ratios, the optimal mobile phase was determined to be the water: methanol: triethylamine: glacial acetic acid (70:28:1:1, v/v/v/v) ratio as it eluted a more prominent peak with characteristics that were desirable for aminophenazone, dipyrine, and chlorpheniramine maleate. This mobile phase gives symmetrical peaks that are clearly defined and very sensitive.

Because three analytes have hydrophobic characteristics, Shim‐pack brands of C18 (4.6 mm × 15 cm; 5 μm) columns were examined. Shim‐pack C18 columns are well suited for hydrophobic analytes. This column has 18 carbon atoms bonded to silica, creating a nonpolar surface that strongly retains hydrophobic compounds. Also, Shim‐pack had a shorter retention time with good theoretical plates. Thus, we selected Shim‐pack brand.

By scanning the wavelength that ranged from 200 to 400 nm, the analyzers’ UV spectrum was recorded. A wavelength of 254 was chosen as the highest wavelength from the spectrum.

### 3.3. Method Validation

#### 3.3.1. System Suitability

The effectiveness of the column determined from aminophenazone, dipyrine and chlorpheniramine maleate peak were found to be 5123, 4655, and 3859 theoretical plates, tailing factor of the aminophenazone, dipyrine and chlorpheniramine maleate peak were found to be 1.23, 1.13, 1.17, and the %RSD of five aminophenazone, dipyrine and chlorpheniramine maleate were found to be 0.10%, 0.06%, and 0.17%, which indicates that the system is suitable for analysis.

#### 3.3.2. Specificity Study

It was observed that after all specific injections, aminophenazone, dipyrine, and chlorpheniramine maleate, gave the same response in sample and standard with respect to retention time and no interference due to diluent and placebo. Therefore, the peak response of the method is considered as specific.

In standard solution, the capacity factor of dipyrine is 2.30 and the aminophenazone is 4.28. Capacity factor between 1 and 5 is considered as good. Selectivity of dipyrine is 1.87 (above 1.1 is considered as good).

Specificity study with RT is presented in Table 3. HPLC chromatogram of diluent, API, standard, and sample solution are given in (Figure 3(a), 3(b), 3(c), 3(d), and 3(e)).

**TABLE 3:** Specificity study.

Name of the solution	Name of peak	Retention time	Diluent/placebo interference
Blank	—	—	—

API	Aminophenazone	15.55	—
	Dipyrine	7.69	—
	Chlorpheniramine maleate	2.08	—

Sample	Aminophenazone	15.92	—
	Dipyrine	7.89	—
	Chlorpheniramine maleate	2.08	—

FIGURE 3Chromatogram of (a) diluent, (b) aminophenzone API, (c) dipyrine API, (d) chlorpheniramine maleate API, and (e) standard solution.

**Figure:**
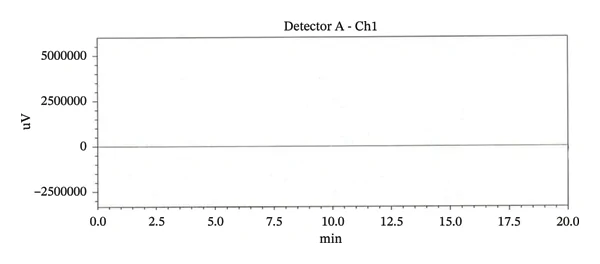
(a)

**Figure:**
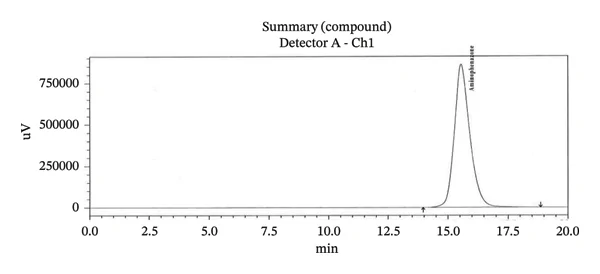
(b)

**Figure:**
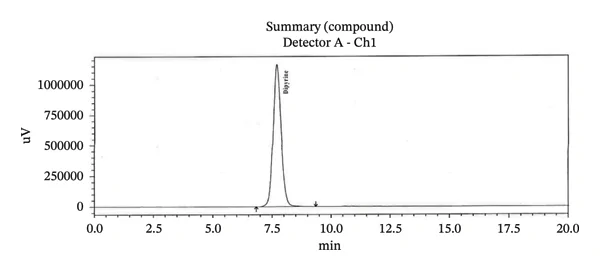
(c)

**Figure:**
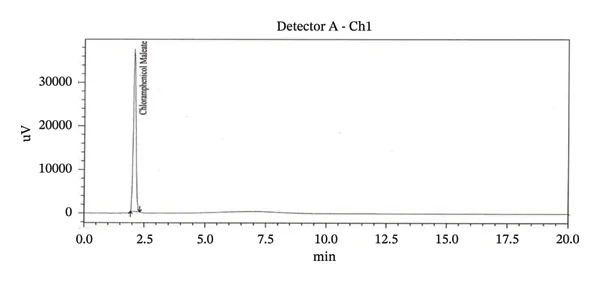
(d)

**Figure:**
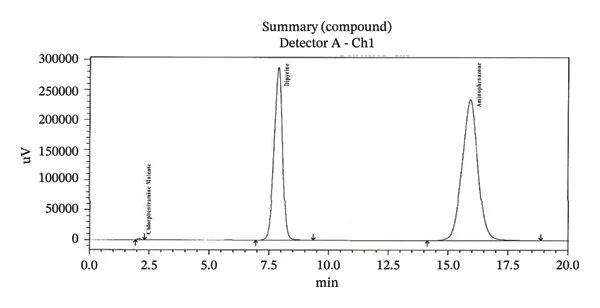
(e)

#### 3.3.3. LOD/LOQ

The value of LOD has been found to be 0.00618 (aminophenazone), 0.02462 (dipyrine), and 0.00015(chlorpheniramine maleate) mg/mL. The LOQ value was found 0.01874 (aminophenazone), 0.07461 (dipyrine), and 0.00045 (chlorpheniramine maleate) mg/mL. S/N ratio of aminophenazone, dipyrine, and chlorpheniramine maleate peaks were within the acceptance limit. The S/N ratio was found to be greater than 3 in the case of LOD and greater than 10 in case of LOQ.

#### 3.3.4. Linearity

**FIGURE 5:**
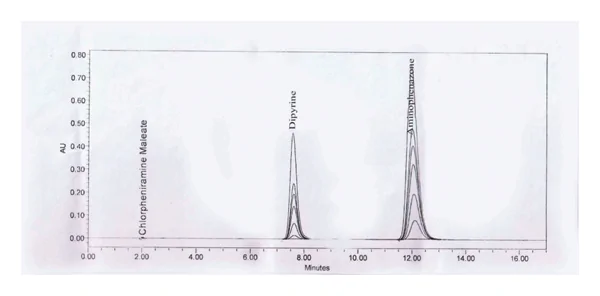
Chromatogram of the linearity study.

The correlation coefficient (*r* ^2^) of aminophenazone, dipyrine, chlorpheniramine maleate were found to be 0.99892, 0.99662, and 0.99992. The results were well within the acceptance limit. Thus, the method is considered linear. Linearity data are presented in Table 4, Figures 4 and 5.

**TABLE 4:** Regression and statistical parameters for the determination of aminophenazone, dipyrine and chlorpheniramine maleate using the proposed HPLC method (n = 6).

Parameters	Aminophenazone	Dipyrine	Chlorpheniramine maleate
Linearity range (mg/mL)	0.1–1.0	0.1–1.0	0.002–0.020
LOD (mg/mL)	0.00618	0.02462	0.00015
LOQ (mg/mL)	0.01874	0.07461	0.00045
Correlation coefficient (r^2^)	0.99892	0.99662	0.99992
Intercept (a)	295723	182471	88
Slope (b)	21691957	15189545	1364705
Standard deviation of intercept (*S*_a_)	303521	343488	81
Standard deviation of slope (*S*_b_)	501363	567382	6725
Standard deviation of residuals (*S*_y/x_)	283614	320959	76
*S* *b* ^2^	25 × 10^10^	32 × 10^10^	45 × 10^6^
%RSD of the slope (Sb%)	2.31	3.73	0.49
(*a*/(*S* *a*))^∗^	0.97	0.53	1.08
*F*	1855	690	40855
Significance F	2.75 × 10^−5^	0.0001	2.67 × 10^−7^

^∗^Theoretical value of t (*a*/*Sa*) = 2.31 at the 95% confidence level.

FIGURE 4Graphical presentation of the linearity study of (a) aminophenzone, (b) dipyrine,and (c) chlorpheniramine maleate.

**Figure:**
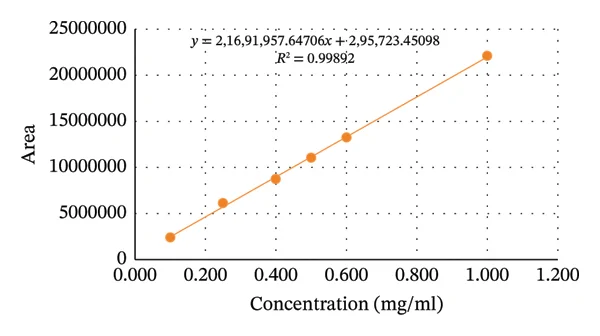
(a)

**Figure:**
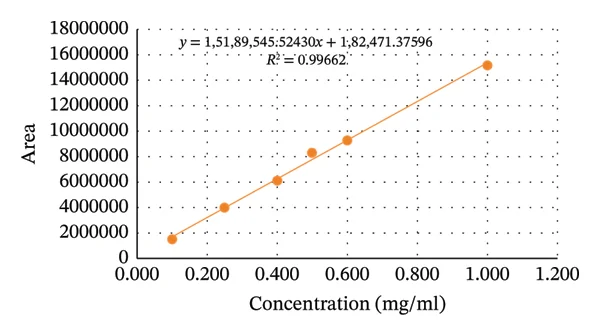
(b)

**Figure:**
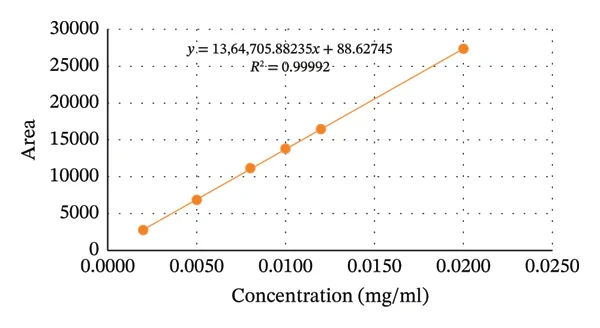
(c)

#### 3.3.5. Precision

Method Precision. %RSD of aminophenazone, dipyrine, and chlorpheniramine maleate were 0.11%, 0.10%, and 0.32% in six sample solutions.

Intermediate Precision. %RSD of aminophenazone, dipyrine, and chlorpheniramine maleate were 0.04%, 0.06%, and 0.41% in six sample solutions.

Cumulative %RSD of aminophenazone, dipyrine and chlorpheniramine maleate were 0.08%, 0.08%, and 0.36% in 12 sample solutions (method precision and intermediate precision). Hence, the method meets the requirement of precision study.

#### 3.3.6. Accuracy

The accuracy in terms of % recovery of four different concentrations was well enough within the acceptance limit (98.00–102.00) %. The %RSD was below 2.0. The accuracy study data are presented in Table 5.

**TABLE 5:** Accuracy study of aminophenazone, dipyrine, and chlorpheniramine maleate recovered.

Name of API	Aminophenazone				Dipyrine				Chlorpheniramine maleate			
	Level of accuracy				Level of accuracy				Level of accuracy			
Sample	LOQ	80%	100%	120%	LOQ	80%	100%	120%	LOQ	80%	100%	120%
1	98.32	98.96	99.10	100.07	98.64	99.56	100.31	99.30	98.43	99.43	100.07	99.55
2	98.45	98.11	99.05	99.19	98.54	99.44	100.12	99.94	99.19	99.74	99.43	100.01
3	98.64	98.65	98.76	99.43	98.69	99.61	100.65	98.65	98.68	99.44	99.70	100.10
Mean	98.47	98.57	98.97	99.56	98.62	99.54	100.36	98.30	98.77	99.54	99.73	99.89
SD	0.16	0.43	0.18	0.45	0.08	0.09	0.27	0.65	0.39	0.18	0.32	0.30
%RSD	0.16	0.44	0.19	0.45	0.08	0.09	0.27	0.65	0.39	0.18	0.32	0.30

#### 3.3.7. Robustness Study

The robust study was satisfactory with column oven temperature (28℃ and 32℃), flow rate (0.8 and 1.2 mL/min), and mobile phase (organic) composition variation (±5%). The %RSD of aminophenazone, dipyrine, and chlorpheniramine maleate peak area were found within the normal ranges. The absolute difference of the content at different modified conditions was found within the range. Details are given in Table 6.

**TABLE 6:** Robustness study (% difference of solution injected at the proposed method and solution injected at the variation method).

Sample name	% difference at different column oven temperature		% difference at different flow rate		% difference at different mobile phase composition (organic, methanol ratio)	
	28℃	32℃	0.8 mL/min	1.2 mL/min	−5%	+5%
Standard solution	0.4%	0.7%	1.1%	1.1%	1.4%	1.6%
Sample solution	0.3%	1.0%	0.9%	1.0%	1.6%	1.8%

#### 3.3.8. Solution Stability Study

The solution was stable up to 24 h as they provided same response as initially provided even if in the room temperature (benchtop), autosampler (15°C–25°C), and refrigerator (2–8°degree) using both transparent and amber vial. After 24 h, the peak area, retention time, tailing factor, theoretical plate, and assay result are found the same as initial result. It can be said that all three actives are stable up to 24 h. The absolute differences from initial to 24 h result are presented in (Table 7).

**TABLE 7:** Solution stability study (difference of initial solution and solution after 24 h).

Parameter	Stability condition (after 24 h)	API	Avg. peak area	% RSD of peak area	RT	Tailing factor	Theoretical plates number	Assay result found	Absolute difference of assay results from original (99.10%)
Standard solution	Room Temperature (benchtop)	Aminophenazone	10320965	0.32	15.65	1.36	5061	—	—
		Dipyrine	7267362	0.53	7.60	1.27	4621	—	—
		^∗^Chlor M	13524	0.61	2.08	1.26	3914	—	—
	Autosampler (15℃ to 25℃)	Aminophenazone	10306602	0.43	15.46	1.37	5058	—	—
		Dipyrine	7245810	0.80	7.60	1.27	4614	—	—
		^∗^Chlor M	13510	0.81	2.04	1.26	3923	—	—
	Refrigerator (autosampler (2℃ to 8℃)	Aminophenazone	10354790	0.65	15.45	1.37	5061	—	—
		Dipyrine	7329831	0.81	7.60	1.27	4621	—	—
		^∗^Chlor M	13574	0.59	2.02	1.25	3965	—	—

Sample solution	Room Temperature (benchtop)	Aminophenazone	10355392	0.24	15.85	1.34	4992	98.33%	0.77
		Dipyrine	7239214	0.43	7.62	1.27	4832	98.70%	0.39
		^∗^Chlor M	13452	0.74	2.07	1.26	3988	98.01%	1.09
	Autosampler (15℃ to 25℃)	Aminophenazone	10293499	0.55	15.29	1.36	5003	98.45%	0.66
		Dipyrine	13309	0.78	7.62	1.27	4785	99.54%	0.44
		^∗^Chlor M	13530	0.73	2.04	1.26	3912	98.09%	1.01
	Refrigerator (Autosampler (2℃ to 8℃)	Aminophenazone	10303435	0.24	15.77	1.34	5011	99.04%	0.06
		Dipyrine	7234091	0.42	7.62	1.28	4602	99.33%	0.23
		^∗^Chlor M	13492	0.71	2.01	1.24	3849	98.54%	0.56

^∗^Chlorpheniramine maleate.

#### 3.3.9. Forced Degradation

The drug product was subjected to stress testing in order to verify the specificity of the analytical methods, induce force degradation, and assess the stability of the drug substance. The analytical technique is capable and trustworthy to show and identify any anticipated changes in the medication product assay throughout stability testing. The stress testing results are summarized in Table 8.

**TABLE 8:** Stress test results.

Stress type	% of degradation (assay)		
	Aminophenazone (%)	Dipyrine	Chlorpheniramine maleate
Acid hydrolysis	11	13	14
Base hydrolysis	9	7	7
Oxidation	24	35	22
Thermal	15	18	30
Photodegradation	No change	No change	No change

### 3.4. Application in Marketed Product

We applied this proposed method in the marketed sample, TD pyrin injection and Samu Pyrin Injection. Assay results have been found in average is satisfactory. Table 9 represents the result found.

**TABLE 9:** Marketed sample analysis report.

Marketed sample with manufacturer	Assay result found (API)		
	Aminophenazone (50 mg/mL)	Dipyrine (50 mg/mL)	Chlorpheniramine Maleate(1 mg/mL)
TD Pyrin Injection (Techno Drugs Limited, Bangladesh)	49.23 mg/mL (98.46%)	49.12 mg/mL (98.24%)	0.99 mg/mL 99.00%
Samu Pyrin Injection (Samu Median Co. Ltd., Korea)	49.53 mg/mL 99.06%	50.80 mg/mL 101.60%	1.01 mg/mL 101.00%

### 3.5. Comparison With Existing Report

A comprehensive comparison of methods shows that the proposed method is suitable for daily analysis. In case of detection limit, majority of the reported methods are not evaluated. The proposed method has a excellent correlation coefficient value. Highly toxic reagents are used in existing methods which are not eco‐friendly. The present method uses only low level of organic and has no complex/tedious procedure. Overall, this approach maybe an environment friendly and not expensive. Table 10 illustrates details of the comparison.

**TABLE 10:** Comparison study of the proposed method with the published method.

Parameters	API	Proposed method	Reported methods
Linearity Range	Aminophenazone	0.1–1.0 mg/mL (100–1000 μg/mL)	1–150 μg/mL [18], 0.5–100 μg/mL [19], 8–8000 ng/mL [20], 102.4–153.6 μg/mL [21],
	Dipyrine	0.1–1.0 mg/mL (100–1000 μg/mL)	4.5–38 μg/mL [22], 4.1–140.0 mg/mL [24], 0.233–600 μg/mL [26], 5.0–35.0 μg/mL [30],
	chlorpheniramine maleate	0.002–0.020 mg/mL (2–20 μg/mL)	0.5–50.0 ng/mL [32], 3.2–4.8 μg/mL [33], 1–50 μg/mL [34], 0.5–2.5 μg/mL [36], 16–24 μg/mL [38], 0.5–2.5 μg/mL [21], 0.01–0.03 mg/mL [39],

Correlation Coefficient (r)	Aminophenazone	0.99892	0.99 [18], 0.9996 [21]
	Dipyrine	0.99662	0.9994 [22], 0.9993 [26], 0.9998 [30],
	chlorpheniramine maleate	0.99992	0.999 [32, 33], 0.9998 [34, 38], 0.997 [36], 0.994 [39],

LOD	Aminophenazone	15.55 mg/mL	0.1 μm/mL [19], 8 ng/mL [20]
	Dipyrine	7.69 mg/mL	1.15 μg/mL [22], 1.2 mg/mL [24], 0.776 μg/mL [26], 0.428 μg/mL [30],
	chlorpheniramine maleate	2.08 mg/mL	0.5 ng/mL [32], 0.0321 μg/mL [36], 32 ng/mL [21],

Diluent	water, methanol, triethylamine, and glacial acetic acid (70:28:1:1 v/v)		0.1 M phosphate buffer [19], 60% MeOH [21], Water: MeOH (50:50; v/v) [22], ACN: MeOH: Water (10:25:65; v/v) [24], 0.01 M KH2PO4− ‐methanol‐acetonitrile‐isopropyl alcohol (420:20:30:30; v/v) [26],0.05 M dibasic phosphate buffer: acetonitrile (93: 07; v/v) [33], ACN: MeOH: phosphate buffer (50: 20: 30; v/v/v; pH 5.6) [36], MeOH: Water (60: 40; v/v) [38] ,MeOH/ammonium dihydrogen phosphate buffer (90:10, v/v) [39],

Retention time	Aminophenazone	15.55	10 min [21],
	Dipyrine	7.69	6.7 min [22], 4.20 min [24], 7.88 min [26],
	chlorpheniramine maleate	2.08	2.74 min [33], 4.213 min [36], 2.00 min [38], 4.20 min [21], 6.8 min [39],

## 4. Conclusion

A simple, affordable, and fast analytical RP‐HPLC‐UV method optimized for simultaneous measuring aminophenazone, dipyrine, and chlorpheniramine maleate in injectable dosage form and validated in accordance with the standard ICH guidelines. The literature review concluded that it is difficult to simultaneously identify aminophenazone, dipyrine, and chlorpheniramine maleate analytically as dipyrine has characteristics to decompose rapidly when it comes to the aqueous solution. Considering decomposition of dipyrine, we optimized the method for inhibiting decomposition reaction, and we successfully achieved it. The findings demonstrated that the standards for robustness, accuracy, precision, linearity, interference, and solution stability were met. It makes sense that this analytical approach can identify aminophenazone, dipyrine, and chlorpheniramine maleate in one single run and that it is also feasible enough to use in pharmaceutical analysis. It would be better to conduct the greenness assessment to this method. In terms of measuring the environmental impact of this analytical technique, green assessment is mandatory. Future prospective would be greenness the method.

## Author Contributions

Conceptualization: Md. Abid Hasan, Tasnuma Tabassum, and Abdul Gafur; literature review: Sajia Azmi, Ejaj Sumit, and Naima Helal; supervision: Md. Abid Hasan and Abdul Gafur; analysis: Tasnuma Tabassum, Sajia Azmi, Ejaj Sumit, Abdul Gafur, and Naima Helal; method optimization and protocol preparation: Md. Abid Hasan; writing manuscript: Md. Abid Hasan, Sajia Azmi, and Ejaj Sumit; review and editing: Md. Abid Hasan, Tasnuma Tabassum, and Naima Helal.

## Funding

This work was done by self‐fund.

## Conflicts of Interest

The authors declare no conflicts of interest.

## Data Availability

The article presents all the results of the analysis. The raw datasets during the study are available upon request.
