# Correction to “Validated RP‐HPLC‐UV Method for Simultaneous Quantification of Aminophenazone, Dipyrine, and Antihistamine in Pharmaceutical Formulations Considering Dipyrine Decomposition”

**DOI:** 10.1155/ianc/9853498

**Published:** 2026-11-10

M. A. Hasan, S. Azmi, T. Tabassum, et al., “Validated RP‐HPLC‐UV Method for Simultaneous Quantification of Aminophenazone, Dipyrine, and Antihistamine in Pharmaceutical Formulations Considering Dipyrine Decomposition,” *International Journal of Analytical Chemistry* 2026 (2026), 7435313, https://doi.org/10.1155/ianc/7435313.

In the article titled “Validated RP‐HPLC‐UV Method for Simultaneous Quantification of Aminophenazone, Dipyrine, and Chlorpheniramine Maleate in Pharmaceutical Formulations Considering Dipyrine Decomposition,” the title was incorrect. Based on the author’s investigation, the method can detect other antihistamines, including cetirizine hydrochloride, brompheniramine, and dexchlorpheniramine. Furthermore, several published studies have demonstrated that a single analytical method can simultaneously detect and quantify multiple antihistamine compounds. References: https://doi.org/10.1155/2019/9489723 https://doi.org/10.1016/j.greeac.2024.100116 https://doi.org/10.1002/jps.2600501011.

The correct title is shown below:

“Validated RP‐HPLC‐UV Method for Simultaneous Quantification of Aminophenazone, Dipyrine, and Antihistamine in Pharmaceutical Formulations Considering Dipyrine Decomposition.”

The online version of this article has been corrected accordingly.

We apologize for this error.

